# Navigating the complex link between infant sleep and development: feels like decoding the Escher labyrinth

**DOI:** 10.1093/sleep/zsae205

**Published:** 2024-09-05

**Authors:** Karen Spruyt

**Affiliations:** Université Paris Cité, INSERM NeuroDiderot, Paris, France

The systematic review titled “The Association Between Infant Sleep, Cognitive, and Psychomotor Development” by Butler et al.(*SLEEP* 2024 in press) [1] critically examines the existing literature on the relationship between sleep in typically developing infants and their cognitive and psychomotor development. Synthesizing findings from 22 studies, the review highlights significant heterogeneity in results, methodologies, and age ranges. This editorial emphasizes the need for developmental reasoning, in addition to standardized methods and replication studies, to unravel the complex link between infant sleep and developmental outcomes. That is, the current scientific findings form a labyrinth reminiscent of M.C. Escher’s (1918–1969) intricate designs.

One of the most notable twists in this labyrinth is the striking heterogeneity in the data across the reviewed studies. According to Butler et al. (*SLEEP* 2024, in press) [1], while two studies found significant associations between sleep and developmental outcomes, 17 reported mixed or three nonsignificant results. This variation stems from several factors, such as the diverse methods used to measure sleep (with eight different assessments and numerous parameters) and developmental outcomes (with 10 cognitive and psychomotor measures). Moreover, the studies employed different assessment time points, with 10 using longitudinal designs. For example, some studies focused exclusively on nocturnal sleep, while others included total sleep duration, encompassing naps. The developmental assessments ranged from evaluating cognitive skills like problem-solving to psychomotor skills such as fine motor coordination. The lack of consistency in both sleep variables and developmental measures complicates the ability to draw definitive conclusions about the relationship between sleep and infant development, especially over the long term. This heterogeneity underscores the urgent need for standardized methodologies at least for certain developmental stages in future research to enable more accurate comparisons and facilitate replication studies.

Another twist in the Escher-like infant sleep labyrinth is the influence of cultural differences. These differences influence sleep habits and their developmental impacts, yet Butler et al.’s (*SLEEP* 2024, in press) [1] review omits discussion on sleep practices or arrangements. Although eight studies employed the Brief Infant Sleep Questionnaire [2], a widely used tool that requires more rigorous psychometric evaluation, few of the reviewed studies address the aspect of sleep practices. Sleep practices can vary widely across cultures, influenced by factors such as co-sleeping habits, parental involvement, and societal norms regarding infant sleep [3, 4]. For example, the safe-to-sleep campaign [5], along with Tummy Time, stresses daytime-prone positioning for psychomotor development, noting that insufficient exposure can delay developmental progress. These cultural variations can lead to different developmental outcomes, complicating the task of comparing results from studies conducted in different cultural contexts. Addressing parental expectations through step-wise culturally sensitive approaches might better meet family needs and improve the nightly rest of the entire family [6, 7].

The next one in Escher’s infant sleep labyrinth is the significant variability in sleep methodologies used across studies. Some studies used objective measures such as polysomnography or (semi-)objective actigraphy to assess sleep patterns, while others relied on subjective measures like parental reports or sleep diaries. Objective measures tend to provide more reliable data, but they are often more costly and difficult to implement on a large scale. For actigraphy, in particular, substantial variability in sleep outcome measures complicates its translation to clinical practice [8]. On the other hand, subjective measures, while easier to collect, are prone to biases and inaccuracies, further contributing to the variability in the findings. Each method faces challenges with the operationalization of sleep variables, such as defining bedtime and rise time in infancy, which contributes to the variability in findings.

Another crucial element in this Escher labyrinth contributing to the heterogeneity of findings is the broad range of ages at which sleep and development were assessed. The review covers infants from 0 to 18 months, a period characterized by rapid and significant changes in sleep patterns and developmental milestones. For example, the developmental implications of sleep in a 3-month-old infant may differ significantly from those in a 15-month-old, given the vast differences in cognitive and motor skills between these ages. Given that developmental trajectories are highly individualized, with milestones reached at different times for each infant, it becomes challenging to establish a consistent relationship between sleep and development, particularly when analyzed at the group level.

To simplify Escher’s labyrinth of infant sleep, Butler et al. (*SLEEP* 2024, in press) [1] employed the National Institutes of Health (NIH) Quality Assessment Tool for Observational Cohort and Cross-Sectional Studies to evaluate the methodological quality, ensuring that only studies of fair to good quality were considered in the synthesis of findings. They also assessed statistical power, finding that all but one study had an estimated power of less than 0.80. Each ensures scientific rigor and helps us understand key findings. The review’s focus on typically developing infants, by excluding infants with neurological, physiological, or genetic disorders, enables controlling for some confounding variables that may skew the results, as discussed in a previous comparable review [9]. However, the exclusion of non-typically developing infants means that the review by Butler et al. (*SLEEP* 2024, in press) [1] does not address the potential moderating effects of developmental disorders on the relationship between sleep and development.

The intricate relationship between sleep and development, akin to Escher’s “Drawing Hands,”(1948) remains elusive. This complexity underscores the necessity for refining research methodologies to address the inherent variability in infant development and highlights the importance of conducting replication studies. For early childhood, future research should particularly include cross-cultural samples to examine how different sleep practices impact developmental outcomes. Achieving clearer scientific insights will not only improve clinical practice but also assist parents in optimizing sleep to support healthy cognitive and psychomotor development in infants.

To date, navigating the labyrinth of infant sleep and development is like deciphering an Escher drawing—intricate, elusive, and endlessly complex. To unravel this enigma, we must apply developmental reasoning, refine our research methods, and embrace replication studies. Only then can we transform the abstract patterns of sleep development into clear, actionable insights that will enhance clinical practice and guide parents in optimizing sleep to promote healthy development.
